# Foot Drop Secondary to Ankle Sprain in Two Paediatric Patients: A Case Series

**DOI:** 10.7759/cureus.26398

**Published:** 2022-06-28

**Authors:** Nikki Shah, Krishna Vemulapalli

**Affiliations:** 1 Trauma and Orthopaedics, Barking, Havering and Redbridge University Hospitals NHS Trust, London, GBR

**Keywords:** paediatrics, ankle sprain, neuropathy, common peroneal nerve, foot drop

## Abstract

Foot drop secondary to common peroneal neuropathy is frequently due to trauma or external compression. Ankle sprains are a rarer cause of this pathology and are extremely uncommon in the paediatric population. We present two cases of acute isolated unilateral foot drop in children, both following minimal trauma. Prompt investigation with magnetic resonance imaging (MRI), electromyography (EMG) and nerve conduction studies can assist in localising the level of the lesion and indicate prognosis. Both patients made a full recovery with the use of ankle-foot orthoses and physiotherapy. This case series highlights that although rare, common peroneal nerve palsy can occur in children following relatively minor trauma. Clinicians should identify this pathology early with a detailed clinical assessment and focussed investigations to increase the potential for a favourable recovery and avoid secondary problems.

## Introduction

Foot drop is caused by a weakness of dorsiflexion of the ankle and toes, leading to a lack of heel strike during ambulation [1,2]. It can be seen in isolation, and it can be unilateral or bilateral and temporary or permanent [2,3]. The most common cause of foot drop is common peroneal neuropathy which can be due to external compression of the common peroneal nerve as it winds around the fibula head, direct trauma or traction injuries [1-3]. Other causes include lower motor neurone disorders such as L5 radiculopathy or lumbosacral plexopathies [1,2]. Central causes, such as amyotrophic lateral sclerosis, are less common but must also be considered [1]. Inversion ankle sprains are extremely common injuries in the general population; however, concomitant injury to the common peroneal nerve resulting in foot drop is rare, and even more so in the paediatric population [4,5]. This diagnosis should not be missed, as it can lead to recurrent falls and subsequent injury [2]. In this article, we present two paediatric cases of unilateral isolated foot drop secondary to common peroneal neuropathy, as a result of minimal trauma. Given the rarity of this condition, this case series is a valuable addition to the limited cases already present in the literature. We also discuss the pathophysiology, diagnosis and management of these injuries. The aim of this article is to highlight the possibility of this injury occurring and the importance of early identification to increase the likelihood of recovery and prevent secondary problems.

## Case presentation

Patient 1 

A 16-year-old male presented with a two-week history of difficulty walking due to an inability to dorsiflex the right ankle. There was no clear traumatic event, but there was some speculation that he may have sustained a minor ankle injury whilst playing football. He denied any associated pain and swelling; however, he was unable to participate in any sporting activities thereafter despite being a keen footballer. He had no significant past medical history to note and does not take any regular medication.

**Figure 1:**
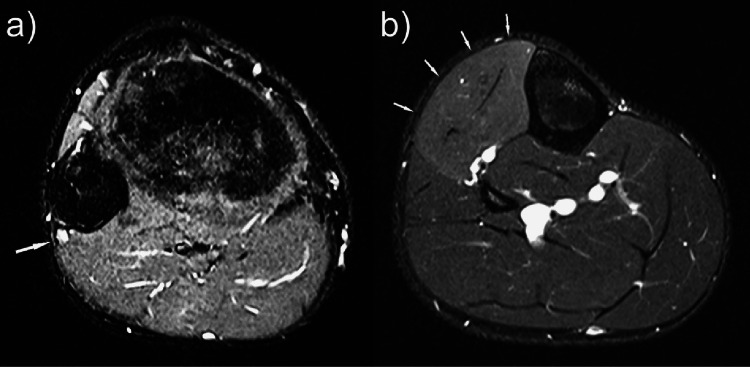
(a) Axial slice of an MRI STIR sequence of patient 1’s right knee demonstrating oedema of the common peroneal nerve as it passes around the fibula head. (b) Axial slice of an MRI STIR sequence of the right calf demonstrating oedema of the anterior compartment consistent with denervation. STIR, short tau inversion recovery

Clinical examination identified a typical foot drop gait. Tibialis anterior (TA), extensor hallucis longus (EHL) and extensor digitorum longus (EDL) were all weak, with a power of 2-3/5 on the Medical Research Council (MRC) scale [6]. Tibialis posterior, flexor hallucis longus and flexor digitorum longus were all working normally with an MRC grade of 5/5. He was able to straight leg raise to over 70 degrees, and the sciatic stretch test was negative. There was no sensory loss throughout the entire foot and ankle and no tenderness over the proximal fibula. Examination of the spine was unremarkable. He was provided with ankle-foot orthoses (AFO) whilst awaiting further investigations and referred for physiotherapy. He underwent a magnetic resonance imaging (MRI) scan of the lower leg which demonstrated mild diffuse oedema and swelling in the anterior compartment of the right leg. There was associated oedema of the common peroneal nerve as it winds around the fibular head, suggestive of low-grade denervation (Figure 1).

Electromyography (EMG) showed fibrillations and positive sharp waves with some polyphasic motor units and reduced interference pattern in the right TA and EHL muscles. Nerve conduction studies involved stimulating various sites in the lower limbs and recording responses at extensor digitorum brevis. There was a drop in amplitude in right lateral popliteal motor responses when stimulated at the knee, 3.3 millivolts (mV) compared to 6.6 mV on the contralateral side). The right superficial peroneal sensory response was smaller in amplitude than that of the left side, 8 microvolts and 13 microvolts, respectively (Table 1). The overall report suggested a mostly neuropraxic lesion but with some axonal loss of the common peroneal nerve at the level of the fibular head. There was evidence of early reinnervation, indicating a favourable recovery.

**Table 1:** Nerve conduction studies for patient 1 comparing the right and left lateral popliteal nerve responses. Various sites in the lower limb were stimulated with responses at the extensor digitorum brevis muscle recorded.

Nerve stimulated	Stimulation site	Record	Latency (m/sec)	Amplitude (millivolt)	Distance (cm)	Velocity (m/sec)	F-wave latency (m/sec)
Right lateral popliteal	Ankle		4.2	10.3	47	46	62
	Below knee	Extensor digitorum brevis					
	Above knee		14.4	3.3			
Left lateral popliteal	Ankle		3.9	8.6	48	46	53
	Below knee	Extensor digitorum brevis					
	Above knee		14.4	6.6			

He was reviewed three weeks later (five weeks from the initial injury) and displayed improvement in dorsiflexion of the great and lesser toes with an MRC grade of 4 out of 5. He was advised to continue with the foot drop splint and physiotherapy and had fully recovered two months following the injury.

Patient 2

**Figure 2:**
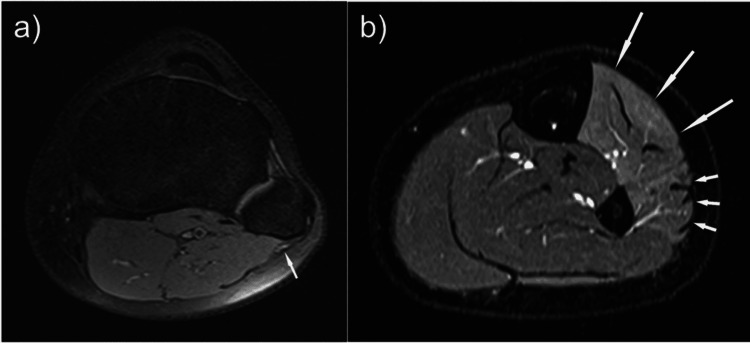
(a) Axial T2 fat-supressed MRI slice of patient 2’s left knee demonstrating swelling of the common peroneal nerve at the fibula head. (b) Axial T2 fat-suppressed MRI slice of the mid-calf demonstrating oedema of the anterior (long arrows) and peroneal (short arrows) compartments.

A 15-year-old female presented with an inability to dorsiflex her left foot and toes. She reported trouble walking with recurrent trips and falls. Five weeks prior to her presentation, she was walking her dog and twisted her ankle. She developed pain immediately after the injury as well as the inability to feel anything below her mid-lower leg. She had a history of asthma. On examination, she displayed a classical foot drop gait with an inability to walk on her heels. She was unable to dorsiflex at the ankle, and her MRC grade of TA was 0/5. EHL and EDL were 1/5 and 2/5, respectively. Sensation was entirely normal despite her initial presentation. There was no tenderness throughout the foot and ankle nor over the proximal fibula. Radiographs of the knee, lower leg and ankle did not reveal any bony abnormality. She was initially treated with an AFO and referred for physiotherapy. An MRI scan revealed mild-to-moderate atrophy of the left anterior and peroneal compartments with associated oedema of the musculature. There was minimal oedema around the common peroneal nerve as it winds around the fibular head, with no evidence of a mass lesion (Figure 2).

EMG studies showed fibrillations and positive sharp waves in EHL and TA. EHL also displayed no recruitment of motor units under voluntary control. TA had a reduced but high firing rate with polyphasic motor units. Nerve conduction studies showed a drop in amplitude of lateral popliteal motor responses at the knee, 0.5 mV on the left compared with 6.9 mV on the contralateral side. Left lateral popliteal motor velocity was also reduced at 38 metres/sec (m/s) compared to 51 m/s on the right (Table 2). Superficial peroneal sensory responses were normal. These findings were consistent with a severe common peroneal nerve lesion, with no signs of reinnervation.

**Table 2:** Nerve conduction studies for patient 2 comparing the right and left lateral popliteal nerve responses. Various sites in the lower limb were stimulated with responses at the extensor digitorum brevis muscle recorded.

Nerve stimulated	Stimulation site	Record	Latency (m/sec)	Amplitude (millivolt)	Distance (cm)	Velocity (m/sec)	F-wave latency (m/sec)
Right lateral popliteal	Ankle		3.6	7.1	40	51	46
	Below knee	Extensor digitorum brevis					
	Above knee		11.4	6.9			
Left lateral popliteal	Ankle		3.8	4.1	42	38	63
	Below knee	Extensor digitorum brevis					
	Above knee		14.7	0.5			

She was reviewed one month later and had some improvement in function with an MRC grade of 3/5 in both EHL and EDL. Power in TA remained 0/5. Due to an improvement in function, she was advised to continue with the use of the AFO and physiotherapy. She regained full function within four months of her initial injury.

## Discussion

Isolated foot drop following minor ankle injury in a paediatric patient is very uncommon and should be identified early to ensure adequate recovery and functional outcome. Foot drop has widely been classified as an MRC grade of <3/5 as this is the inability of the foot to dorsiflex against gravity [3]. This leads to a ‘steppage’ or ‘slapping’ gait with hip and knee flexion to allow the foot to clear the floor during the swing phase of the gait cycle [2,3,7]. In the long term, this can result in muscle imbalance, which leads to shortening of the Achilles tendon and development of an equinus foot position [3]. Foot drop can therefore lead to recurrent tripping or falling, increasing morbidity. Along with weakness in foot dorsiflexion and extension of the toes, other signs and symptoms may be present, depending on the underlying cause [2]. It is therefore essential to undertake a thorough history and examination to assist in diagnosis, especially as a delay in presentation is possible [4,5,8]. Whilst foot drop has many aetiologies, injury to the common peroneal nerve is the most frequent cause of this condition [1]. When there is a complete lesion, there is an inability to dorsiflex the foot and extend the toes, as well as weakness in foot eversion [1,4,7]. Sensory loss is classically over the anterolateral surface of the lower leg and the dorsum of the foot [1,2,7]. The location of the lesion, severity and duration of injury can dictate the clinical findings. The involvement of the individual fascicles of the common peroneal nerve can also lead to a variable presentation in motor and sensory signs, posing a diagnostic challenge [1]. The close proximity of the common peroneal nerve to the neck of the fibula makes it vulnerable to trauma or external compression. After exiting the popliteal fossa, it passes through a tendinous tunnel, between the edge of the peroneus longus muscle and the fibula [1,7]. At this level, it lies directly on bone and is only covered by skin and subcutaneous tissue. Pressure effects from habitual leg crossing, prolonged kneeling or squatting, tight plaster casts and masses such as tumours are all well-known causes of foot drop [1]. Ankle sprains causing acute foot drop are a much rarer cause, but not unheard of. Several theories have been put forward explaining the mechanism in which acute ankle sprains can result in common peroneal neuropathy. Oppenheim was the first to describe traction of the common peroneal nerve during sudden forcible supination of the foot [9]. A study by Hyslop suggested that anatomical variation predisposing to the stretching effect on the common peroneal nerve or direct pressure of the forceful contraction of the overlying muscles near the neck of the fibula contributes to the neuropathy following an inversion ankle sprain [10]. Nobel suggested that torsional injuries of the ankle could rupture the vas nervorum of the common peroneal nerve, forming a haematoma within the nerve sheath. This could also present as a delayed foot drop due to a developing and expanding haematoma [11]. In the two cases described by Nobel, prompt evacuation of the haematoma and ligation of the vas nervorum was carried out, leading to swift recovery. An ankle sprain complicated by common peroneal neuropathy is primarily seen in adults, and only a small handful of cases in the paediatric group have been documented [5,8]. Garozzo et al. reported a case of a 13-year-old girl who sustained a common peroneal neuropathy following an ankle inversion injury whilst playing basketball [8]. This patient developed pain and swelling in the ankle immediately after the injury, which is similar to most of the other cases reported in the literature in which ankle sprains cause a common peroneal neuropathy. She underwent surgical intervention following failed recovery with non-operative measures.

In both our patients, minor trauma was sustained with no visible signs immediately following the injury, apart from minimal pain in patient 2’s case. This is extremely rare, even more so in children, and has only been described once by Wang et al. [5]. This article describes a case of a nine-year-old boy who sustained a minor ankle sprain with no evidence of immediate pain or swelling following the injury. He showed a full recovery following non-operative management with an AFO, which is identical to our two cases. Both of our patients underwent prompt investigation with MRI and electrodiagnostic tests within weeks of presenting to outpatient clinic. Although the first-line test in lesion localisation in peripheral nerve lesions is electrodiagnostic testing, MRI has been proven to improve accuracy in diagnosing peripheral nerve lesions and be a valuable adjunct to electrodiagnostic tests [12,13]. Nerve conduction studies commonly show a low amplitude and/or conduction block across the fibular head, which was seen in both our cases [4]. Similarly, both patients had the presence of positive waves and fibrillation action potentials at rest, which indicate denervation [14]. Patient 1 showed a mostly neuropraxic lesion with some axonal loss, which generally has a favourable recovery, whereas patient 2 displayed a more severe common peroneal nerve lesion. Non-operative and surgical management have been described for common peroneal nerve palsy as a result of ankle sprain. Brief et al. advise surgical decompression and neurolysis in patients who show no improvement following four to five weeks following ankle injury [7]. A systematic review concludes that surgery is indicated within three to seven months from injury if there is no evidence of improvement following the injury, but is less successful if surgery is delayed by more than six months [4]. Our patients showed improvement in function within one month of presentation following provision of an AFO and physiotherapy. An AFO supports the foot whilst walking, facilitates mobilisation and reduces the risk of falling [1,2,4,7]. Targeted physiotherapy can prevent muscle atrophy, secondary muscle tightness and preserve ankle mobility [3,4]. The significant improvement seen in our patients within several weeks creates a stronger argument for continuing with non-operative management, and both have made a full recovery.

## Conclusions

Common peroneal neuropathy resulting in acute foot drop secondary to a minor ankle sprain is extremely rare in the paediatric population. Foot drop should be identified early to avoid severe functional impairment. A thorough history and examination avoids the need for extensive testing and facilitates targeted investigations and management. Treatment is tailored according to the underlying cause, but initial management includes the provision of an AFO and physiotherapy. For patients who do not respond to these treatments, surgical exploration and decompression of the common peroneal nerve can be considered.
